# Study on Deterioration Law and Mechanism of Gray Brick Due to Salt Crystallization

**DOI:** 10.3390/ma15082936

**Published:** 2022-04-18

**Authors:** Jianwei Yue, Yuan Li, Zhenxian Luo, Xuanjia Huang, Qingmei Kong, Zifa Wang

**Affiliations:** 1 School of Civil Engineering and Architecture, Henan University, Kaifeng 475004, China; yjw@vip.henu.edu.cn (J.Y.); liy@henu.edu.cn (Y.L.); hxj_henu@126.com (X.H.); 10160004@vip.henu.edu.cn (Q.K.); zifa@iem.ac.cn (Z.W.); 2 Key Laboratory for Restoration and Safety Evaluation of Immovable Cultural Relics, Kaifeng 475004, China; 3 Yellow River Civilization and Sustainable Development Research Center, Henan University, Kaifeng 475004, China

**Keywords:** salinization, gray brick, crystallization pressure, deterioration mechanism

## Abstract

Salinization has an important impact on the degradation of ancient masonry buildings, and systematically mastering the law of salt migration and degradation of ancient masonry buildings is an important part of the protection of ancient buildings. In this paper, the damage law of gray bricks under the action of salt crystallization is studied. The orthogonal test method is used to carry out cyclic degradation tests on gray bricks. The nominal strength is proposed as a mechanical parameter to measure the structural damage of grey bricks, and the change in compressive strength and crystallization pressure of the samples after the test is measured and analyzed. The results show that the damage of different salts in the gray bricks shows a certain difference. Magnesium sulfate and sodium chloride cause significant damage to the surface of the gray bricks, while calcium chloride does not cause significant damage to the surface of the gray bricks. When the concentrations of sodium chloride solution, calcium chloride solution and magnesium sulfate solution are less than 13.73 mol/L, 11.47 mol/L and 17 mol/L, respectively, the nominal strength of gray brick samples increases; In the range of 9.9 mol/L and 4.73–8.94 mol/L, the crystallization pressure began to appear inside the sample. The research results provide an important scientific basis for evaluating the damage caused by salting to the damage of porous ancient building materials such as masonry.

## 1. Introduction

Ancient buildings have numerous characteristics that include large volume, a complex occurrence environment, immovability, and a vulnerability to environmental influence. With the passage of time, various physical and chemical changes of ancient buildings lead to diseases such as peeling, pulverization and cracking of brick masonry. The effect of salinity is the main problem affecting the durability of porous building materials, and it has become an indicator to measure the durability of porous building materials [1,2,3]. Studying the presence and behavior of salt in porous building materials has become the focus of international research [4,5,6,7,8,9,10]. The growth of salt crystals in confined spaces (pores) can exert a pressure sufficient to exceed the tensile strength of most stones and alter their porosity, pore size distribution, and mechanical properties [11,12,13]. Different materials will have different damage conditions under the action of the same salt, and Benavente et al. [14] summarized the factors affecting salt weathering in porous materials, such as pore structure and porosity, transport properties of salt solutions in pores, material strength, and resistance to crystallization pressure. As a porous medium material fired from clay, brick is widely used in various ancient buildings and architectural sites. Therefore, it is very important to study the migration and deterioration mechanism of salt in bricks for the preventive protection of ancient brick buildings [15,16,17,18].

Water absorption and salt crystallization are the most common problems and the most serious phenomena leading to the degradation of ancient masonry buildings. Soluble salt is the main deterioration factor of porous building materials such as masonry and will reduce its strength. Water, as a transport tool for other substances such as soluble salt, transports soluble salt to different locations of the material to crystallize, so the moisture transport properties of the material have a great influence on the location of salt crystallization [19]. The growth of salt crystals in the pores of building materials can generate enough stress to overcome their tensile force, and the degradation of many ancient buildings can be attributed to soluble salts. Çelik [20] studied the effect of different salt solutions and their different concentrations on the capillary water absorption of a Turkish limestone, the kind of limestone shows a bimodal pore size distribution with considerable amounts of micropores and especially unimodal pore size distributions. The results show that micropores have shown particularly susceptibility to salt crystallization. When the salt solution concentration is low, the salt content in the limestone pores is not enough to fill the pores, causing damage and increasing its uniaxial compressive strength.

Menéndez et al. [21] studied the effect of different factors, brine composition, environmental conditions and pore structure on the salt weathering of porous carbonate building stone, and performed wetting and drying cycles with different relative humidity on samples containing salt. Its results show that single salt brines, especially sodium sulphate, produce more weathering than complex brine compositions during wetting and drying cycles. In historic buildings, capillary rise of soil water is one of the main sources of salt in the structure, which evaporates leaving behind salt. The spatial distribution of different types of salt crystallized in historic buildings is not uniform, i.e., different types of salt crystallize in different locations. Marques et al. [22] conducted an experimental study on the change of sandstone porosity caused by salt crystallization, and evaluated the compressive mechanical behavior of sandstone during the aging test of sodium chloride crystallization, the results show that the difference in compressive strength values between monotonic and cyclic compression also decreases, a predictive equation is proposed that relates the compressive strength of sandstone to the salt crystallization aging cycle. The crystallization pressure generated during the evaporation and crystallization of salt solution is the main cause of damage to building materials [23]. For brittle porous media, the size and distribution of crystallization pressure between pore walls mainly depend on the type of crystal and pore morphology. Ju et al. [24] generalized various complex pore morphologies into four ideal pore morphologies, including cylindrical shape, and deduced the calculation formula for the size and distribution of the pore wall and inter-crystal pressure during crystal formation.

To sum up, scholars at home and abroad have carried out extensive research on the damage of porous media materials caused by salt crystallization, however, they have mainly focused on rock building materials such as sandstone and limestone. In order to find out the deterioration rule of gray bricks caused by salt crystallization, this paper conducts salt crystallization tests on gray bricks based on the orthogonal experiment method. In the salt crystallization test, sodium chloride, calcium chloride, and magnesium sulfate solutions were used, and the solution concentrations were 1%, 3%, and 5%. The macroscopic physical and mechanical properties of gray bricks (such as saturated water absorption, compressive strength, nominal strength) and the change law of the microstructure of gray bricks after salt crystallization, were obtained on this basis, and the deterioration mechanism of gray bricks under the action of crystallization pressure generated by salt crystallization was analyzed. The research results have important practical significance for the preventive protection of ancient masonry buildings, and provide a certain basis for evaluating the damage of salt to porous ancient buildings such as masonry.

## 2. Materials and Methods

### 2.1. Experiment Material

#### 2.1.1. Gray Brick

The gray bricks used in the test are those left by the demolition of ancient dwellings in Kaifeng City, Henan Province. In order to reduce the influence of the discrete nature of the material on the test, gray bricks with similar colors and a difference of ±3% in dry weight were selected, and the samples were cut by a cutting machine into a 5 cm × 5 cm × 10 cm cuboid. The sample was washed in deionized water about 10 times to reduce the influence of the existing salt in the pores of the sample on the capillary cycle test, and then the washed gray brick sample was dried to a constant weight and stored in a desiccator for use.

**Figure 1:**
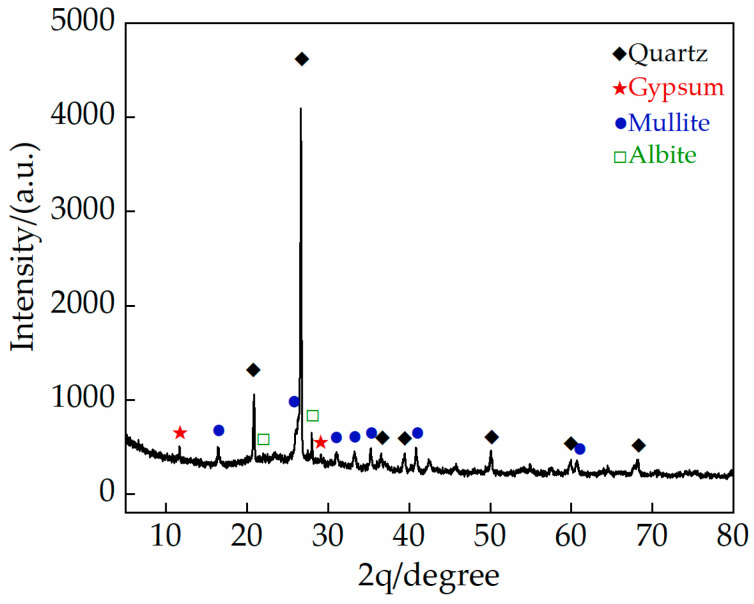
X-ray diffraction (XRD) patterns of original gray brick.

**Figure 2:**
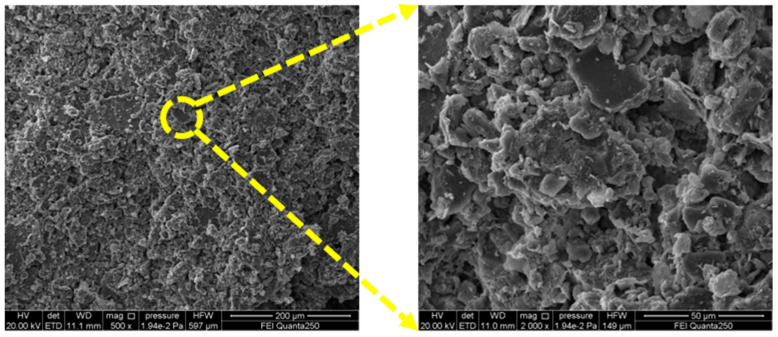
Microscopic SEM image of untested gray brick sample.

Before carrying out the test, the gray bricks are firstly subjected to characterization tests, such as chemical, phase analysis (SEM, XRD, XRF analysis) and physical properties for material characterization. The main chemical components of gray bricks measured by X-ray fluorescence spectrometer (Rigaku ZSX Primus; Rigaku, Akishima, Japan) are shown in Table 1, and are mainly composed of silicon dioxide (SiO_2_), aluminum oxide (Al_2_O_3_), calcium oxide (CaO), iron oxide (Fe_2_O_3_), potassium oxide (K_2_O) and other metal oxides. In addition to the main components, gray bricks also contain trace amounts of titanium dioxide (TiO_2_), phosphorus pentoxide (P_2_O_5_) and so on. The mineral composition of the green bricks was analyzed by X-ray diffractometer (Rigaku D/max 2400; Rigaku, Akishima, Japan) and the results are shown in Figure 1, which indicate that the main components of the bricks are composed of gypsum, mullite and sodium feldspar. In addition, the original samples were observed with a scanning electron microscope (JEOL JEM-F200; JEOL, Akishima, Japan) and the image results are shown in Figure 2. The porosity of the grey brick samples was determined by a Micromeritics Auto Pore V 9600 instrument (Micromeritics; Norcross, GA, USA). According to the relevant specifications and requirements [25], an electro-hydraulic servo testing machine (MTS 810; MTS, Eden Prairie, MN, USA) was used to test the compressive strength and flexural strength of the ash bricks, and the experimental loading speed was 0.1 mm/s. Three specimens were tested for each index and the average value was taken as the final value, the physical and mechanical parameters of grey bricks are shown in Table 2.

#### 2.1.2. Type and Concentration of Salt

(1) Choice of soluble salt. The salt used in the test is classified as sodium chloride, calcium chloride and magnesium sulfate, according to the characteristic table of chemical composition of groundwater in Kaifeng City [26]. The cations in surface water are mainly Na^+^, Ca^2+^, Mg^2+^, accounting for 43.6%, 36.1% and 20.3% of the total content, respectively. The anions are dominated by HCO_3_^−^, accounting for 71.7% of the total, followed by SO_4_^2−^, accounting for 16.8%, and the content of Cl^−^ is the lowest, accounting for only 11.4% changes. The types of anions and cations in the groundwater are the same as those in the surface water, only their content varies.
(2) Determination of salt solution concentration. According to the characteristics table of groundwater chemical components in Kaifeng City [26] and the XRF test results of salt frost collected from the surface of the north side of Kaifeng City Wall at different heights, 1%, 3%, and 5% were finally set as the test salt solution concentration values.

### 2.2. Cyclic Deterioration Test

The specific test steps are as follows: ① Stack two permeable stones with a diameter of 10 cm and a thickness of 1 cm at the bottom of each sample; ② Add salt solution to make the liquid level equal to the top of the permeable stone; ③ Take out the sample when the sample absorbs the solution for 30 min; ④ Put the sample, after inhaling the salt solution, into a drying oven at 95 °C for 3 h, which is a cycle test. According to the meteorological data of Kaifeng City: from 2000 to 2020, the annual average number of times the daily temperatures in Kaifeng City exceeded 35 °C was 13.28, and three cycle times gradients of 10 times, 20 times, and 30 times were set.

In order to find out the influence of various factors on the deterioration of gray bricks, the soluble salt type (A), the salt solution concentration (B) and the number of cycles (C) are used as the three test indicators of the orthogonal test group in this paper. A three-factor and three-level orthogonal experiment was designed (Table 3), and pure water was set as the control group, with 30 cycles for a total of 10 experimental groups, and 6 parallel samples were set in each group. Each sample was tested 5 times per cycle and averaged.

### 2.3. Nominal Strength Test

**Figure 3:**
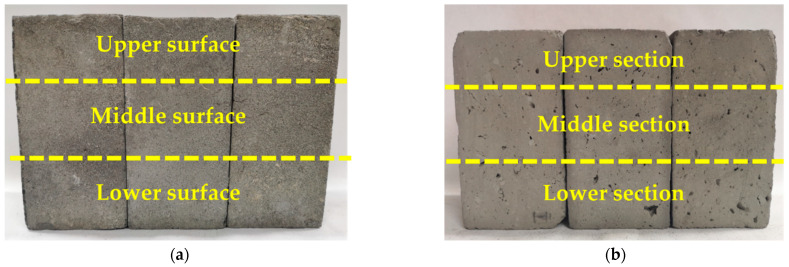
Diagram of the different test areas for the nominal strength of the sample: (**a**) the surface of the sample; (**b**) the cutting surface of the sample.

In order to quantify the extent of the damage caused to the brick surface by salt crystallization during the test, this paper uses nominal strength to characterize the mechanical properties of the sample surface, dividing the specimen surface and the cut surface into three areas (Figure 3)—top, middle and bottom—and testing the sample after each cycle using an Eidelberg HP-500 push–pull gauge. The test procedure is as follows: the sample is placed on the push–pull table and the value is recorded when the 1.5 cm diameter circular probe is lowered to the surface of the sample and produces a reading of approximately 2–3 N. Four parallel samples are tested in each group and the average value is taken and the nominal strength is calculated using Equation (1).

(1) $$P=\frac{F}{S\times 1,000,000}$$

where *P* is the nominal strength of the sample (MPa), *F* is the pressure on the sample (N), and *S* is the surface area of the push–pull gauge probe (mm^2^).

### 2.4. Compressive Strength Test

After the cyclic deterioration test was completed, the compressive strength of the grey brick samples was tested using an electro-hydraulic servo tester (MTS 810) with a test loading speed of 0.1 mm/s. Three samples were selected from each experimental group and the average value was taken as the final compressive strength.

## 3. Results and Discussion

### 3.1. Changes in Sample Appearance and Quality Due to Salt Crystallization

**Figure 4:**
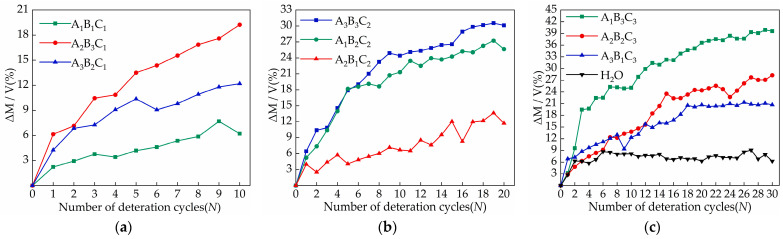
The influence curve of different salt content and cycle times on sample quality: (**a**) A_1_B_1_C_1_, A_2_B_3_C_1_, A_3_B_2_C_1_; (**b**) A_3_B_3_C_2_, A_1_B_2_C_2_, A_2_B_1_C_2_ ;(**c**) A_1_B_3_C_3_, A_2_B_2_C_3_, A_3_B_1_C_3_, H_2_O control group.

In this paper, the relative mass of each sample in the same volume is analyzed to reduce the deviation of the initial mass to test results caused by different manufacturing processes. Figure 4 is the R curve of the sample mass growth rate, and the growth rate is the ratio of the relative change of mass to the volume, as shown in Equation (2).

(2) $$R=\frac{M_{t}-M_{0}}{V}\times 100\%$$

where *M*_0_ is the initial mass of the sample (g), *M_t_* is the sample mass after the *N*th cycle (g), and *V* is the sample volume (mm^3^).

In the cyclic deterioration test, the sample is absorbed by capillary action for 30 min in a salt solution and then dried in a drying oven at 95 °C for 3 h. The sample is cooled to room temperature after drying and is then weighed to measure the sample mass. There are usually two issues associated to the change in quality; salt crystallization inside the sample that leads to an increase in sample mass, and salt crystallization close to the sample surface that causes the sample surface to exfoliate, resulting in a decrease in sample mass. The final effect depends on the soluble salt species and salt solution concentration. In this experiment, the mass of the samples increased, and the concentration of the salt solution was the main factor affecting the proportion of the increase in the mass of the samples, and it was also the key to determine the damage status of the samples. From the analysis of Figure 4, 5% calcium chloride (A_2_B_3_C_1_) resulted in the largest increase in sample mass (19.25%) over 10 cycles. Similarly, in the samples of 20 and 30 cycles, the magnesium sulfate (A_3_B_3_C_2_) and sodium chloride (A_1_B_3_C_3_) groups with 5% salt solution concentration increased the most in mass, at 30.12% and 39.58%, respectively.

The samples usually increased rapidly in the first six cycles. From the seventh cycle, the quality of the control group began to remain stable, and the growth rate of the samples in the experimental group decreased. This is due to the evaporation of water not destroying the internal pores of the sample, and drying at 95 °C for 3 h means that the sample cannot be completely dried, and the residual water remains in the pores. With the accumulation of soluble salt crystals, the crystallization pressure causes the microcracks inside the sample to expand, and the solution enters the internal pores, while the increase in salt content and the expansion of pores also increases the water absorption of the sample. In addition, sodium chloride will cause the surface of the sample to form a shell and peel away from the sample, and the surface of the sample containing magnesium sulfate will crack and gradually pulverize.

### 3.2. The Variation in the Uniaxial Compression Strength Values Due to Salt Crystallization

All the gray brick samples were damaged after the test, the deterioration caused by magnesium sulfate was the most serious, and the damage caused by sodium chloride to the gray brick samples was the second highest. It can be seen from Table 4 that the 5% magnesium sulfate (13.1 MPa) after 20 cycles and the 3% magnesium sulfate (14 MPa) gray brick samples cycled 10 times have the largest loss of compressive strength. Calcium chloride caused the least decrease in the compressive strength of gray bricks. However, the saturated water absorption results of the gray brick samples are opposite to the compressive strength. The saturated water absorption of the magnesium sulfate test samples increased the least (21.73% on average), and the saturated water absorption increased the most for calcium chloride (23.16%). The salt crystallization tests carried out showed that the hydration of the salt crystals resulted in microcracks within the material and slight changes in the surface micromorphology and strength reduction at different salt solutions and concentrations. These microcracks were also observed in SEM inspection of all samples.

### 3.3. Change in Nominal Strength

**Figure 5:**
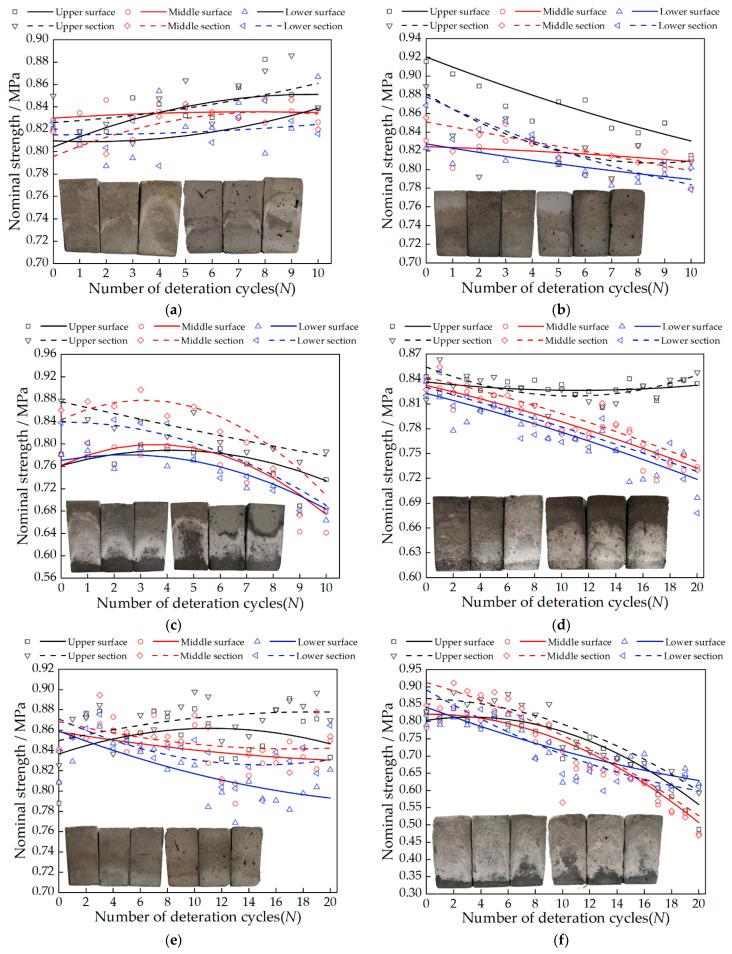

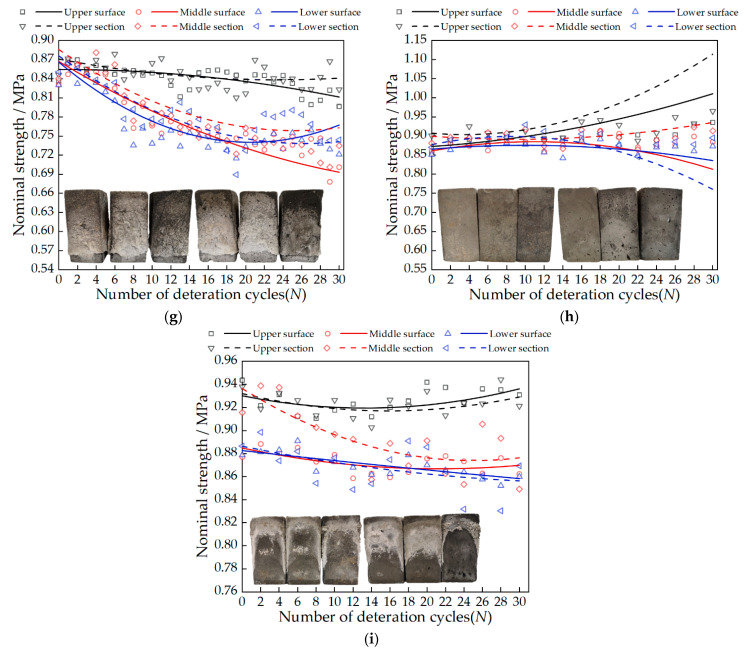
Fitting curve of nominal strength of different test samples: (**a**) A_1_B_1_C_1_; (**b**) A_2_B_3_C_1_; (**c**) A_3_B_2_C_1_; (**d**) A_1_B_2_C_2_; (**e**) A_2_B_1_C_2_; (**f**) A_3_B_3_C_2_; (**g**) A_1_B_3_C_3_; (**h**) A_2_B_2_C_3_; (**i**) A_3_B_1_C_3_.

Origin software was used to fit the test data. Figure 5 shows the nominal strength fitting curves of the surface and section of the samples in each test group. The results show that the change of the nominal strength of the sample is closely related to the type of salt. Among the three salts, 20 cycles of 5% magnesium sulfate (A_3_B_3_C_2_) caused the largest decrease in the nominal strength of the samples (0.27 MPa on average), followed by 20 cycles of 3% sodium chloride (A_1_B_2_C_2_), and the average nominal intensity of the samples was down by 0.12 MPa. Calcium chloride caused the least damage to the samples, with 20 cycles of 1% calcium chloride and 30 cycles of 3% calcium chloride samples with an increase in the upper nominal strength. Soluble salt crystallization is an important deterioration mechanism of gray bricks, the damage is caused by the crystallization pressure applied in the pores, and the microcracks caused by this mechanism can be observed in SEM.

**Figure 6:**
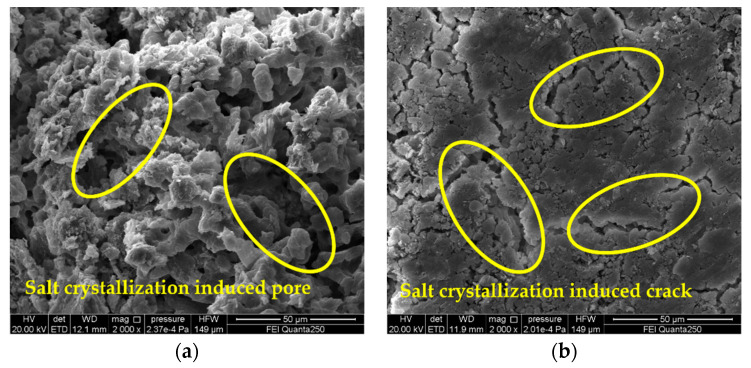
SEM image of gray brick sample of 5% magnesium sulfate (A_3_B_3_C_2_) cycled 20 times. Detail of microcracks induced by crystallization of magnesium sulfate salt (yellow circle). (**a**) inside the sample; (**b**) the surface of the sample.

**Figure 7:**
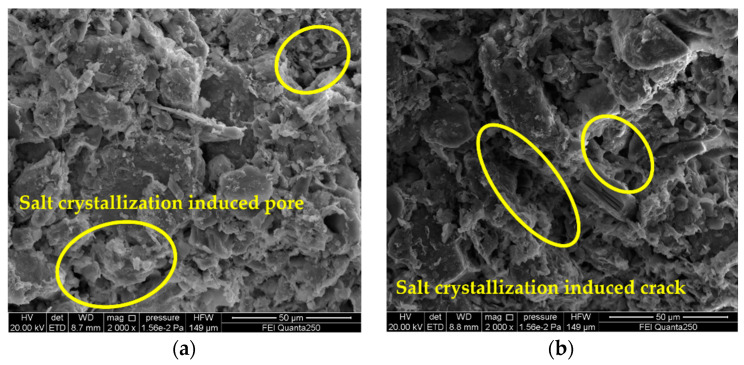
SEM image of gray brick sample of 3% sodium chloride (A_1_B_2_C_2_) cycled 20 times. Detail of sodium chloride salt crystallization induced micro fissure (yellow circle). (**a**) inside the sample; (**b**) the surface of the sample.

**Figure 8:**
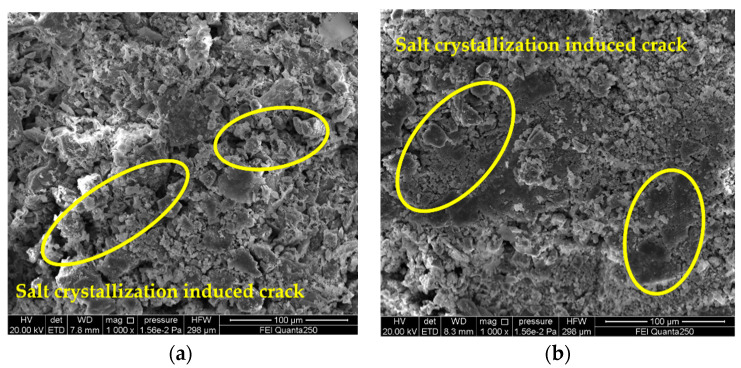
SEM image of gray brick sample of 3% calcium chloride (A_2_B_2_C_3_) cycled 30 times. Detail of microcracks induced by calcium chloride salt crystallization (yellow circles). (**a**) inside the sample; (**b**) the surface of the sample.

Figure 6 presents the SEM image of the 5% magnesium sulfate sample (A_3_B_3_C_2_) cycled 20 times and the details of the microcracks induced by the salt crystallization. SEM observation showed that the gray brick samples were severely damaged by sulfate crystallization, and microcracks were generated during the cycle test. Subsequently, the crystals continued to grow and deposit in the cracks that had already formed, and the microcracks caused the crack enlargement and chalking phenomena observed macroscopically on the surface of the gray brick samples. Figure 7 presents the SEM images and details of the salt crystallization-induced microcracks of the gray brick samples after 20 cycles of the salt crystallization test with 3% sodium chloride (A_1_B_2_C_2_). It is found that the sodium chloride is mainly deposited in the microcracks formed by salt crystallization, which were also found on the mineral surface and in the pores. Figure 8 presents the SEM images and details of the salt crystallization-induced microcracks of the gray brick samples after 30 cycles of the salt crystallization test of 3% calcium chloride (A_2_B_2_C_3_), and it can be seen that the calcium chloride crystals are quite different from the other two salt crystals. The calcium chloride crystals are smaller in size and mainly deposited in the pores of the minerals, which not only did not cause more microcracks in the gray brick samples, but also affected the pores. It plays a filling role and improves the compactness of the sample.

To sum up, the damage to the tiny cracks of gray bricks by magnesium sulfate crystals is the most serious, and the damage of tiny pores is the key to the damage of salt crystals [27], so the nominal strength of the samples in the A_3_B_3_C_2_ group decreased the most. This confirms the conclusion of Lindström et al. [28] that magnesium sulfate has a high damage potential at high temperature. However, it is different from Steiger’s [29,30] view that salt first fills larger pores and then fills micro-pores. The difference is mainly due to the different test conditions. The increase in temperature will lead to a corresponding increase in the solubility of the solution, and the saturated solution will turn into an unsaturated solution; evaporation will continuously lead to the concentration of the solution. In this way, the solution entering the pores of the sample gradually converges and crystallizes in the micro-cracks with strong water holding capacity during the concentration process, and causes extrusion damage to both sides of the wall crack, resulting in the expansion of micro-cracks [31].

In addition, the damage status of the upper part of the sample caused by the action of different types of salts is also quite different. There are two main reasons: (1) The saturated solubility of calcium chloride and magnesium sulfate increases with the increase in temperature. When the sample is dried in the drying oven, the solubility of the solution inside the sample increases due to the increase in temperature, and the crystallization rate slows down. With the increase in the number of cycles, it continues to migrate to the upper part along the capillary pores and gradually accumulate, causing extrusion damage to the sample pores. However, the saturated solubility of sodium chloride is not affected by temperature, and the dimension has little effect on the upper part of the sample. (2) When the concentration of the salt solution inside the sample is low, stable crystals will first form in the larger pores, and then the crystals will enter the smaller pores with the increase in the solution concentration. The crystals filled in the larger pores substantially improve the compactness of the sample, although they eventually lead to damage inside the sample, which ultimately reduces the compressive strength of the brick [32].

### 3.4. Analysis on Deterioration Mechanism of Gray Brick Caused by Salt Crystallization

In the capillary cycle test, the salt solution enters the pores of the sample through capillary action. As the water contained in the sample evaporates, the salt concentration in the sample gradually increases. When the ratio of the solubility to the saturated concentration of the solution at the current temperature and pressure (when the supersaturation concentration) is greater than one, it will start to crystallize in the pores to generate crystallization pressure [33]. Therefore, the crystallization pressure generated during the cycle can be calculated through the change in the concentration of the salt solution, and finally the critical point and concentration value, at which the sample begins to destroy under the action of salt can be determined. The equation for calculating the crystallization pressure is as follows [34]:

(3) $$m_{N}=M_{N}-M_{0}$$

where $m_{N}$ is the salt mass in the *N*th cycle sample (g), *N* is the number of cycles, and $m_{RN}$ is the mass of the solution in the *N*th cycle sample (g):

(4) $$w=(\sum_{i=1}^{N}m_{N})/m_{RN}$$

where *w* is the mass fraction of the solution inside the sample after each immersion (%), *N* is the number of cycles, and $m_{RN}$ is the mass of the solution in the *N*th cycle sample (g).

The amount $n_{N}$ of the solute in the solution is:

(5) $$n_{N}=(m_{RN}w)/M$$

where $n_{N}$ is the amount of substance in the solution during the *N*th cycle (mol), *M* is the relative molecular mass of the salt (g/mol). 

The concentration of the solution in the current cycle is:

(6) $$C_{N}=1000m_{N}/M$$

where $C_{N}$ is the concentration of the salt solution inside the sample during the *N*th cycle (mol/L).

The supersaturation of the solution is:

(7) $$\sigma_{N}=(C_{N}-C_{0})/C_{0}$$

where $\sigma_{N}$ is the supersaturation of the solution during the *N*th cycle, $C_{0}$ is the saturated concentration of the solution at room temperature 28 °C (mol/L).

Substituting Equation (5) into Equation (6), the crystallization pressure generated by the crystallization of the salt solution during the current cycle can be obtained:

(8) $$P_{N}=R_{g}Tln\sigma_{N}/\nu$$

where $P_{N}$ is the crystallization pressure (MPa), $R_{g}$ is the gas constant, taken as 8.314 J/(mol·K), *T* is absolute temperature (K), and v is the molar volume of the crystal (L/mol).

**Figure 9:**
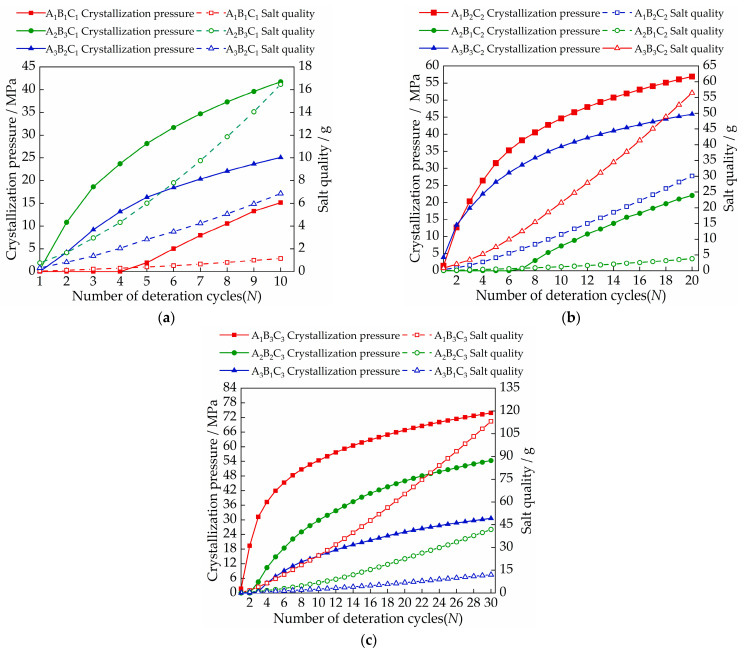
The internal crystallization pressure of each brick sample under different salt content and cycle times, where the salt quality refers to the mass of the solute in the solution inside the brick sample: (**a**) A_1_B_1_C_1_, A_2_B_3_C_1_, A_3_B_2_C_1_; (**b**) A_3_B_3_C_2_, A_1_B_2_C_2_, A_2_B_1_C_2_; (**c**) A_1_B_3_C_3_, A_2_B_2_C_3_, A_3_B_1_C_3_.

The calculated crystallization pressure value is shown in Figure 9. As can be seen from Figure 9, the samples of the A_1_B_1_C_1_, A_2_B_1_C_2_, and A_3_B_2_C_1_ groups had lower crystallization pressures during the first three cycles. With the increase in the number of cycles, the crystallization pressures (Figure 9) began to be generated and gradually increased, and the nominal strengths showed a different degree of improvement. This is due to the solution inside the sample in the early stage having not yet reached the supersaturated state, and with the accumulated salt crystals being less, the crystallization pressure generated by the crystallization of the salt solution is low enough to destroy the pores of the sample. At the same time, the salt crystallization induced by evaporation will have obvious effects on the pore structure. The pore size distribution and porosity of the material are significantly reduced [35]. After calculation, it can be obtained that when the concentrations of sodium chloride solution, calcium chloride solution and magnesium sulfate solution are less than 13.73 mol/L, 11.47 mol/L and 17 mol/L, respectively, the nominal strength of the sample increases. The samples of the highest concentration in the A_2_B_3_C_1_, A_3_B_3_C_2_, and A_1_B_3_C_3_ groups produced a larger crystallization pressure from the beginning of the test, After three cycles, the crystallization pressure of these three groups of samples can reach 18.65 MPa, 20.35 MPa, 31.2 MPa, respectively. At this time, the nominal strength of the samples (Figure 5) began to decrease. With the increase in the number of cycles, the maximum salt crystallization pressure can reach 41.7 MPa, 57 MPa, and 73.87 MPa, respectively. Larger crystallization pressure will inevitably cause damage to the tiny pores inside the brick, crack expansion, and a decrease in compressive strength (Table 5) [36]. After calculation, it can be seen that when the concentrations of sodium chloride solution, calcium chloride solution, and magnesium sulfate solution are in the range of 8.17–8.93 mol/L, 6.98–9.9 mol/L, and 4.73–8.94 mol/L, respectively, crystallization pressure will begin to occur.

It is worth noting that the nominal strength (Figure 5) and compressive strength (Table 5) of the samples in the A_3_B_3_C_2_ group decreased the most, by 0.27 MPa and 4.9 MPa, respectively, and the maximum crystallization pressure was 57 MPa. However, the nominal strength (Figure 5) and compressive strength (Table 5) of the A_1_B_3_C_3_ group samples with a maximum crystallization pressure of 73.87 MPa decreased by 0.12 MPa and 1.6 MPa, respectively. It can be found in Figure 6 and Figure 7 that the microcracks caused by salt crystallization in A_3_B_3_C_2_ are significantly more than those in the A_1_B_2_C_2_ samples, indicating that the degree of sample deterioration is not only determined by the size of the crystallization pressure, but also related to the distribution of salt crystals in the pores of different sizes of the sample [37]. Derluyn et al. [38] developed a fully coupled computational model that describes the deformation and damage of porous materials due to heat, water and salt ion transport, crystallization stress, and a computational model of deformation and damage that simulates sodium chloride crystallization damage to porous limestone. The simulations show that the effective stresses resulting from salt crystallization do not only depend on the crystallization pressure, which is related to the supersaturation, but also on the amount of salt crystals forming and the localization of these crystals.

In practical engineering, the saturated solubility of calcium chloride and magnesium sulfate will increase with the increase in temperature. Therefore, the actual value of crystallization pressure is lower than the theoretical calculation value, but the theoretical calculation value provides a reference for judging the damage of gray brick crystallization, and can be used as a limit reference for judging the damage caused by salt crystallization to an ancient gray brick masonry building’s value.

## 4. Conclusions

Salt crystallization has an important influence on the deterioration characteristics of the ancient brick masonry building body. In this paper, based on the orthogonal test method, the cyclic deterioration test of gray bricks under the action of different types of salt is carried out. The pressure and deterioration laws are discussed, and the microstructure damage characteristics under the action of different salt crystallization are discussed in combination with SEM images. The main conclusions are as follows:

1. The concentration of salt solution is the main factor affecting the relative change in sample quality. Samples of the A_2_B_3_C_1_, A_3_B_3_C_2_, and A_1_B_3_C_3_ groups with a salt concentration of 5% increased the most, which were 19.25%, 30.12%, and 39.58%, respectively. In appearance, the damage to the middle and lower part of the sample is more serious when the sodium chloride solution acts on the sample. With the increase in the number of cycles, the surface will form crusts and fall off. The damage of the magnesium sulfate solution to the upper part of the sample is more significant, mainly including pulverization, cracks, and causing parts to fall off. The calcium chloride solution has no obvious damage characteristics on the surface of the sample;
2. There are two factors that affect nominal strength: salt solution concentration and salt type. Nominal strength decreases with increasing salt solution concentration. Magnesium sulfate crystals have the most serious damage to the tiny pores of gray bricks, followed by sodium chloride and calcium chloride, which has the least. The nominal strength of the samples in the A_3_B_3_C_2_ group decreased the most, with an average decrease of 0.27 MPa. When the concentrations of sodium chloride solution, calcium chloride solution and magnesium sulfate solution are less than 13.73 mol/L, 11.47 mol/L and 17 mol/L, respectively, the nominal strength of the sample increases;
3. The size of the crystallization pressure has a lot to do with the quality of the salt inside the brick. When the concentrations of sodium chloride solution, calcium chloride solution and magnesium sulfate solution are in the range of 8.17~8.93 mol/L, 6.98~9.9 mol/L, and 4.73~8.94 mol/L, respectively, the crystallization pressure begins to occur;
4. By observing the SEM images, it is found that the degree of sample deterioration caused by salt crystallization not only depends on the size of the crystallization pressure, but is also related to the distribution of salt crystals in the pores of different sizes of the sample. In order to better understand the damage mechanism of brick masonry caused by salinization under actual conditions, the next research topics include conducting mixed-salt cyclic deterioration experiments and determining the pore size of the crystalline distribution of different types of salts.

## Figures and Tables

**Table 1:** Main chemical composition of gray brick.

Compound	SiO_2_	Al_2_O_3_	CaO	Fe_2_O_3_	K_2_O	MgO	Na_2_O	TiO_2_
Concentration (%)	62.29	15.63	6.69	5.23	3.74	3.20	1.95	0.68

**Table 2:** Parameters of the grey bricks.

Density/(kg/m^3^)	Poisson’s Ratio	Porosity /%	Compressive Strength/MPa	Elastic Modulus/GPa	Elastic Modulus (GPa)
1800	0.15	15.63	19.27	3.74	3.34

**Table 3:** Factors and levels of orthogonal test.

Test No.	Variable		
	Salt (A)	Salt Concentration (B)	Cycles (C)
A_1_B_1_C_1_	NaCl	1%	10
A_1_B_2_C_2_	NaCl	3%	20
A_1_B_3_C_3_	NaCl	5%	30
A_2_B_2_C_3_	CaCl_2_	3%	30
A_2_B_3_C_1_	CaCl_2_	5%	10
A_2_B_1_C_2_	CaCl_2_	1%	20
A_3_B_3_C_2_	MgSO_4_	5%	20
A_3_B_1_C_3_	MgSO_4_	1%	30
A_3_B_2_C_1_	MgSO_4_	3%	10

**Table 4:** Results of orthogonal tests.

Test No.	Salt	Salt Concentration/%	Cycle	Saturated Water Absorption/%	Compressive Strength/MPa
0	H_2_O	0	30	20.4	18
1	NaCl	1%	10	21.2	15.7
2	NaCl	3%	20	22.6	15.3
3	NaCl	5%	30	24.4	16.4
4	CaCl_2_	3%	30	23	16.7
5	CaCl_2_	5%	10	23.8	16
6	CaCl_2_	1%	20	22.7	17.4
7	MgSO_4_	5%	20	22.3	13.1
8	MgSO_4_	1%	30	21	15.5
9	MgSO_4_	3%	10	21.9	14

**Table 5:** Results of orthogonal tests.

Test No.	Salt	Salt Concentration/%	Cycle	Saturated Water Absorption/%	Compressive Strength/MPa
0	H_2_O	0	30	20.4	18
1	NaCl	1%	10	21.2	15.7
2	NaCl	3%	20	22.6	15.3
3	NaCl	5%	30	24.4	16.4
4	CaCl_2_	3%	30	23	16.7
5	CaCl_2_	5%	10	23.8	16
6	CaCl_2_	1%	20	22.7	17.4
7	MgSO_4_	5%	20	22.3	13.1
8	MgSO_4_	1%	30	21	15.5
9	MgSO_4_	3%	10	21.9	14

## Data Availability

The data presented in this study are available on request from the corresponding author.
